# The Role of Disgust Emotion in Eating Disorders and Its Relationship with Dissociative Symptoms

**DOI:** 10.3390/healthcare13080954

**Published:** 2025-04-21

**Authors:** María F. Rabito-Alcón, Anabel González-Vázquez, José I. Baile

**Affiliations:** 1 Department of Psychology and Health, Open University of Madrid (UDIMA), 28400 Madrid, Spain; joseignacio.baile@udima.es; 2 Department of Psychiatry, University Hospital of A Coruña, As Xubias S/N., 15006 A Coruña, Spain; anabel.gonzalez.vazquez@gmail.com

**Keywords:** disgust, self-disgust, dissociation, eating disorders, obesity

## Abstract

**Background**: Disgust is a central emotion in eating disorders, influencing both their development and maintenance. High sensitivity to disgust has been associated with restrictive and purgative behaviors, as well as with greater severity of eating disorder symptoms. Despite its importance, the different aspects of disgust are rarely examined in depth, limiting the understanding of its role in these disorders. Since the emotion of disgust is more closely linked to the body and food than other emotions, the aim of this paper is to understand its possible role in eating disorders and obesity. **Method**: In a case–control study, 313 women (155 with eating disorders and obesity in the clinical group and 158 in the control group) were assessed using various questionnaires, including measures of disgust, dissociation, and symptom severity. **Results**: The results showed that the clinical group reported greater symptom severity, more dissociative symptoms, and higher sensitivity to disgust than the control group. It is noteworthy that there were significant differences between subtypes of the clinical group, particularly in disgust related to hygiene, sexual content and deterioration and disease. **Conclusions:** These findings highlight the importance of considering disgust in the evaluation and treatment of eating disorders, emphasizing the need for further research on this emotion to develop more effective therapeutic interventions that address this complex emotion in the context of eating disorders and obesity.

## 1. Introduction

Eating disorders (EDs) are complex disorders characterized by an excessive concern with weight, body shape, and food [1]. For decades, research has focused on the underlying mechanisms of these disorders due to their complexity, as current treatments for EDs remain unsatisfactory in many cases, and these disorders can persist for many years [2,3]. Previous studies have concluded that factors such as dissociation and disgust play significant roles in EDs [4,5] and obesity [6], suggesting a possible link between dissociation and disgust as mechanisms contributing to the risk and maintenance of eating pathology.

Obesity is not considered an eating disorder per se, but its association with certain pathological eating behaviors highlights the importance of addressing this condition in the context of eating disorders. Research indicates that a considerable proportion of individuals with obesity meet the diagnostic criteria for Binge Eating Disorder (BED), which is characterized by recurrent episodes of excessive food intake accompanied by a sense of lack of control, in the absence of compensatory behaviors [7]. BED represents the most prevalent eating disorder among individuals with obesity and is associated with greater psychological distress, poorer quality of life, and more severe clinical outcomes compared to obesity without binge eating episodes. Therefore, considering the overlap between obesity and BED is essential to understand the complex relationship between eating pathology, emotional factors, and weight-related issues.

Disgust is a fundamental human emotion that has evolved as a defense strategy against pathogens and dangerous substances. It manifests as an intense emotional response, often accompanied by physical sensations such as nausea, that motivates avoidance behaviors to reduce the risk of contamination or harm [8,9]. Although it is a basic emotion, its expression and triggers can vary significantly depending on cultural, social, and individual factors, such as gender, age, and life experiences [10]. Disgust can be divided into different types, such as sexual disgust and moral disgust, each serving a specific purpose: protecting genetic integrity and maintaining social norms [10]. Additionally, disgust influences cognitive processes, shaping attention and memory, and has a significant impact on social interactions [10,11,12,13,14].

Disgust and disgust sensitivity have been recognized as individual factors that may influence the etiology of various disorders [15], including specific phobias, such as blood and animal phobias [16,17], hypochondriasis [18], obsessive-compulsive disorder (OCD) [19,20,21], and EDs [2,22], suggesting its role as a transdiagnostic psychopathological construct [2,22,23,24,25]. A distinctive feature of disgust is its heterogeneous nature, activated in response to a variety of stimuli, such as objects, situations, odors, and behaviors. This highlights the importance of evaluating disgust experiences by considering the different types or dimensions that may underlie this emotion [15].

Some authors [15,26] consider disgust to be composed of six basic dimensions: hygiene, moral, sexual, body transgression, animals, and impairment/disease. Table 1 contains information associated with the six basic dimensions.

As indicated earlier, emotions such as disgust have been considered to be involved in problematic avoidance behaviors (e.g., avoid talking about a past issue to avoid experiencing emotional discomfort) and/or compensatory behaviors (e.g., vomiting) frequently observed in EDs [15]. Specifically, many of these studies on disgust in EDs have focused on self-disgust [2,31,32]. Self-disgust can be defined as the emotion that arises when an evaluation of the internal world or physical characteristics is made to be shameful [2]. This self-disgust has been associated with avoidance strategies towards certain parts of oneself that are not considered legitimate according to prevailing beauty standards, such as thinness in the case of women, which may lead, for example, to dietary restriction or hiding different parts of the body. However, disgust is a complex emotion that goes beyond self-disgust.

Evidence on the relationship between disgust and EDs supports the hypothesis that individuals with these disorders exhibit high levels of disgust sensitivity [5,33]. This sensitivity may influence aversive conditioning processes, increase distress, and facilitate feelings of fear and anxiety, maintain core psychopathology, and influence food avoidance behaviors [5,24]. Furthermore, disgust plays a role in generating anxiety and distress in various psychopathologies, as it is involved in complex emotions such as shame and guilt, generating a biased interpretation of threatening situations [24]. Research indicates that individuals with obesity demonstrate lower disgust sensitivity compared to their lean counterparts, as evidenced by functional MRI studies showing reduced activation in the insula when viewing contaminated food images [34] (This diminished disgust sensitivity may contribute to overeating behaviors, as individuals may not adequately respond to food-related disgust cues [34].

Research indicates that people with eating disorders may present dissociative symptoms as a coping mechanism to manage affective states, deal with unresolved trauma, and separate themselves from eating disorder symptoms [35,36,37]. Some authors [38,39,40,41] propose two categories for classifying dissociation: detachment and compartmentalization. Detachment is an altered state of consciousness characterized by a sense of separation from oneself or the environment, including depersonalization and out-of-body experiences. Compartmentalization is associated with a loss of control over processes usually under conscious control, such as the inability to recall information [35,36,37]. Examples include dissociative amnesia, conversion symptoms, and dissociative identity disorder [38,39]. Dissociation may also help manage emotions related to disgust [42]. These patients may use eating disorder-related behaviors, such as purging or binge eating, as a way to induce dissociative symptoms to avoid or escape from feelings, sensations, memories, and cognitions related to traumatic events. In some cases, compensatory behaviors, such as vomiting, are often linked to feelings of disgust and the pursuit of purity resulting from, for example, having experienced sexual abuse [36,43,44]. Various studies in the literature suggest that people with eating disorders, in addition to experiencing a frequent disconnection with their own body, experience sexuality with strangeness and discomfort. In addition, self-induced malnutrition serves, among other functions, to reduce sexual interest and attenuate the development of secondary sexual characteristics in adulthood [45,46]. Therefore, there is an interest in clarifying the underlying mechanisms of eating psychopathology to develop more specific and personalized treatments. Research indicates that obese patients with BED exhibit higher levels of dissociation compared to those without BED, suggesting a complex interplay between psychological factors and obesity [47].

A review of the published works on the subject seems to show a connection between the emotion of disgust and eating disorders, but this relationship is still unclear. The aim of our study is to explore disgust sensitivity and the various aspects related to the emotion of disgust. These include disgust related to hygiene, morality, sexuality, body transgression, animals, and impairment or disease, as well as disgust at a global level. We also seek to determine whether disgust is linked to dissociative symptoms, such as detachment and compartmentalization, and its relationship with severity in women with different subtypes of eating disorders and obesity. Finally, we compare these findings with a group of women without an eating disorder diagnosis to gain a deeper understanding of how disgust, as a complex phenomenon, is involved in eating disorders.

The following hypotheses are proposed in this study:

1. Disgust sensitivity positively correlates with dissociative symptomatology (compartmentalization and detachment) and severity.
2. Greater global disgust sensitivity is expected in the clinical group compared to the control group.
3. Greater dissociation (both detachment and compartmentalization) is expected in the clinical group compared to the control group.
4. Significant differences in overall disgust sensitivity are anticipated across ED subtypes, with variations across dimensions of disgust.
5. Statistically significant differences are expected in dissociation (both detachment and compartmentalization) and severity among ED subtypes and obesity.

## 2. Materials and Methods

### 2.1. Participants and Procedure

This case–control study recruited 305 women between September 2022 and July 2024: 147 patients from various hospitals and psychological centers specializing in EDs in differents regions across Spain diagnosed with eating disorders according to the DSM-IV-TR [48] and DSM-5 criteria [49] or obesity based on the BMI, and 158 women from the general population without eating disorders and obesity, accessed through social media outreach. In the clinical sample, diagnoses included 20% with anorexia nervosa (AN, N = 31), 16.13% with bulimia nervosa (BN, N = 25), 23.87% with binge eating disorder (BED, N = 37), 25.8% with eating disorder not otherwise specified (EDNOS, N = 40) and 9% with obesity (N = 14). Due to the low prevalence of men diagnosed with eating disorders in the participating centers, only women were included. Each participant provided informed consent after being informed of the study’s objectives, in line with the Declaration of Helsinki, and approval was obtained from the Ethics, Research, and Teaching Committee of the Madrid Open University.

Participants in the clinical group were previously diagnosed by their psychiatrist. In each participating center, the therapists of patients diagnosed with eating disorders or obesity were responsible for providing the patients with the link between the form and the study questionnaires. The control group was matched in age and gender to ensure homogeneity. Sociodemographic data, including age, weight, height, type of eating disorder diagnosis, disorder duration, hospitalizations, suicidal ideation, and self-injurious behaviors, were collected using a dataset specifically designed for the study through a form. To check that there was no risk of the women in the general population suffering any type of eating disorder, the EAT-26 was administered. Self-reported anthropometric measurements were also requested, since there are studies [50,51] that have shown that there are no significant differences between self-reported and real anthropometric measurements, to check the BMI of the participants. The main sociodemographic characteristics of the clinical group can be seen in Table 2. Eligibility criteria for the clinical group included women over 18 diagnosed with an eating disorder or obesity, while the control group consisted of women over 18 with no known diagnosis of eating disorders or obesity.

### 2.2. Measurement Instruments

We used the following questionnaires: the Eating Attitudes Test (EAT-26), the Detachment and Compartmentalization Inventory (DCI), the Multidimensional Disgust Sensitivity Scale (EMA), and the Clinical Global Impressions Severity Scale (CGI-S).

EAT-26 [52,53] is a self-report questionnaire consisting of 26 items. The items are rated on a 6-point Likert scale ranging from 1 (“never”) to 6 (“always”). The total score is obtained by recording the responses as follows. Scores of 1 to 3 are recorded as 0, 4 as 1, 5 as 2, and 6 as 3. The only exception is item 25, where responses are scored as follows: 1 as 3, 2 as 2, 3 as 1, and 4 to 6 as 0. The total EAT-26 score ranges from 0 to 78. It demonstrates high internal consistency, generally with Cronbach’s alpha coefficients above 0.90. The EAT-26 is a useful tool for the early detection of eating disorders. Additionally, the EAT-26 is a validated scale for the Spanish population [53]; it is self-administered and straightforward to apply, making it suitable for use in various contexts without the need for direct intervention by a professional. It has been used in multiple studies [54,55,56] across different populations, including adolescents and adults, and in various cultural contexts, supporting its applicability and utility in diverse samples.

DCI [38,39] is a self-report inventory with 22 items, specifically designed to measure experiences of dissociative detachment (10 items, e.g., “What I see seems ‘flat’ or ‘lifeless’, as if I were looking at a picture”) and compartmentalization (10 items, e.g., “I don’t feel like I have control over what my body does, as if there is someone or something inside me directing my actions”) when not under the influence of alcohol or drugs. Additionally, it includes two items to assess response validity (e.g., “I cross the street where there is no crosswalk, i.e., I cross recklessly”). Each item is rated on an eight-point Likert scale measuring the frequency of these experiences (0: never, 7: daily). The DCI is a validated scale for the Spanish population [39], suitable for detecting experiences of dissociative detachment and compartmentalization in both clinical and research settings. The DCI scores also show adequate internal consistency (ranging between 0.87 and 0.90) and test-retest reliability, suggesting it is a reliable scale that also allows for temporal tracking with repeated measures over time [38,39].

EMA [15] is a scale consisting of 30 items that assesses sensitivity or vulnerability to respond with disgust to six different types of triggers through 6 subscales of 5 items each: (1) hygiene, (2) moral, (3) sexual, (4) bodily transgression, (5) animals, and (6) impairment/disease. Participants rate the degree of revulsion each item evokes, ranging from “not at all” (0) to “extremely” (4). In addition to scores on the six subscales, a total score on the scale can also be obtained. The EMA is a validated scale for the Spanish population [15]. Compared to other instruments assessing disgust, the EMA has two main advantages; it is more appropriate for use in Spanish-speaking and Hispanic populations, as other disgust scales are predominantly designed for English-speaking and North American cultural contexts, and it allows for the assessment of basic dimensions of disgust, whereas other questionnaires generally measure only more general dimensions [15]. The EMA has demonstrated excellent content validity (factorial validity), and appropriate levels of reliability (internal consistency and temporal stability), as well as convergent and discriminant validity [15].

CGI-S [57] is a scale that evaluates the clinician’s global impression of the severity of the patient’s illness at the time of assessment. It consists of eight items ranging from 0 (“not assessed”) to 7 (“among the most extremely ill patients”). Although the CGI-S is not evaluated in terms of internal consistency due to its single-item nature, it is considered a reliable tool based on its high inter-rater and test-retest reliability [58]. The CGI-S has been adapted and validated in various contexts, including among Spanish-speaking populations [58].

### 2.3. Statistical Analysis

Data were presented as mean (M) and standard deviation (SD) for continuous variables, while categorical variables were expressed as frequencies and percentages (%). Descriptive statistics were computed for all study variables. Parametric tests were used for the overall sample, and non-parametric tests were applied to comparisons between subtypes of eating disorders, as normality assumptions were not met in these subgroups.

Correlations between variables were assessed to explore relationships within the data. For comparisons between subtypes of eating disorders, the Kruskal–Wallis H test was used due to its robustness against non-normal distributions. The Student’s T-test was employed to compare the clinical group with the control group, given the parametric nature of the overall sample. All analyses were conducted using SPSS for Windows, version 24.0 (IBM Corporation, Armonk, NY, USA), and statistical significance was established at *p* ≤ 0.050.

## 3. Results

### 3.1. Sociodemographic Data

The clinical group had an average age of 35.28 years (SD = 9.70), ranging from 18 to 65 years, while the control group had an average age of 37.97 years (SD = 11.02), ranging from 18 to 63 years. The mean estimated weight was 69.28 kg (SD = 20.32) in the clinical group and 63.82 kg (SD = 12.31) in the control group. Average heights were 164.30 cm (SD = 6.25) in the clinical group and 163 cm (SD = 6.27) in the control group.

Among the participants with eating disorders, the average duration of the disorder was 13.02 years (SD = 8.96), and the mean number of hospitalizations was 1.06 (SD = 3.49). Regarding mental health challenges, 52.8% of the clinical group reported experiencing suicidal ideation, and 61.3% engaged in self-injurious behaviors without intent to end their life. In contrast, 38.6% of women in the control group reported suicidal ideation, and 24.1% reported non-suicidal self-injurious behaviors.

### 3.2. Pearson Correlations Between Disgust Sensitivity and Dissociative Symptoms of Detachment and Compartmentalization, and Severity

Table 3 and Table 4 show the correlations between disgust sensitivity variables (EMA scores) and the other study variables, including dissociative symptoms (DCI scores) and severity (CGI-S score) for each group.

It can be observed in the clinical group that the variables of disgust sensitivity tend to positively correlate with symptoms of dissociation, including detachment and compartmentalization, across all dimensions except for the dimensions of bodily transgression, impairment/disease (only in detachment) and animal-related disgust. Regarding dissociative symptoms, the correlations were positive and significant: 0.33 ** for detachment, 0.28 ** for compartmentalization, and 0.31 ** for total dissociative symptoms. 

### 3.3. Differences Between the Control Group and the Clinical Group in Relation to Disgust Sensitivity and Various Aspects of Disgust, Dissociative Compartmentalization Symptoms and Detachment Symptoms, and Severity

To test our hypotheses regarding differences between the clinical and control groups in disgust sensitivity, dissociation, and severity, we conducted independent samples T-tests. Results showed significantly higher disgust sensitivity in the clinical group (*p* < 0.001). Specifically, the clinical group exhibited higher levels of disgust related to hygiene (*p* < 0.001), sexual content (*p* < 0.001), animals (*p* < 0.007) and impairment/disease (*p* < 0.001) with a moderate effect size. No significant differences were found in moral disgust, bodily transgression, or animal-related disgust. Further analysis confirmed higher dissociation levels in the clinical group, both overall and in specific dimensions of detachment and compartmentalization (*p* < 0.000) with high effect size. The values obtained indicate good internal consistency in both groups. The Cronbach’s alpha coefficient of 0.768 in the control group suggests that the scale items are reasonably consistent with each other, while the value of 0.777 in the clinical group indicates slightly higher internal consistency. These results suggest that the instruments used in the study are reliable for measuring the construct of interest in both populations. Table 5 summarizes the differences between groups in terms of disgust sensitivity, various disgust aspects, dissociation, and severity, including effect size.

### 3.4. Different Subtypes of Eating Disorders and Obesity and Their Relationship with Disgust Sensitivity and Various Aspects of Disgust Emotion, Dissociative Symptoms of Compartmentalization, Dissociative Symptoms of Detachment, and Severity

To test hypotheses 4 and 5 and examine the relationship between different subtypes of eating disorders and disgust sensitivity, we conducted a Kruskal–Wallis H test. This analysis revealed an asymptotic significance of 0.002 (χ^2^ = 20.78; df = 6; *p* = 0.002), indicating statistically significant differences among the various eating disorder subtypes and obesity patients in terms of disgust sensitivity (0.002 < 0.005). Specifically, women diagnosed with anorexia nervosa exhibited the highest levels of overall disgust sensitivity (M = 61.25), while those diagnosed with bulimia nervosa showed the lowest levels (M = 51.82).

Using the same test, we analyzed different dimensions of disgust emotion. The results indicated an asymptotic significance of 0.015 (χ^2^ = 15.72; df = 6; *p* < 0.001) for disgust related to hygiene, of 0.007 (χ^2^ = 17.71; df = 6; *p* < 0.001) for disgust related to sexuality and of 0.001 (χ^2^ = 22.56; df = 6; *p* < 0.001) for disgust related to impairment and disease, with significant differences found in these dimensions. However, no significant differences were observed for disgust associated with morality, bodily transgression, and animals. The highest levels of disgust related to sexuality were found in women diagnosed with obesity (M = 11.28), while the lowest levels were in women with bulimia nervosa (M = 7.48). The highest levels of disgust related to hygiene and deterioration and disease were found in women diagnosed with binge eating disorder (M = 12.08 and M = 7.37, respectively), while the lowest levels were in women with bulimia nervosa (M = 10.36 and 5.08, respectively).

To explore the relationship between different eating disorder subtypes, obesity and dissociative symptoms, we performed another Kruskal–Wallis test. Statistically significant differences were found among eating disorder subtypes regarding dissociative symptoms related to detachment (χ^2^ = 55.91; df = 6; *p* < 0.001) and compartmentalization (χ^2^ = 83.15; df = 6; *p* < 0.001), as well as in the overall level of dissociation (χ^2^ = 71.98; df = 6; *p* < 0.001). These results confirm the fifth preliminary hypothesis of this study, which anticipated significant differences in dissociative symptomatology across different eating disorder subtypes. Specifically, higher dissociation scores, both in detachment and compartmentalization, as well as overall, were observed in the group of women diagnosed with anorexia nervosa, while the lowest levels were found in the obesity group. This supports the possibility that restrictive eating disorder subtypes may be associated with higher levels of dissociation.

Additionally, we expected to find statistically significant differences among eating disorder subtypes regarding severity. Differences were indeed found for this variable as well (χ^2^ = 23.11; df = 6; *p* < 0.001).

Table 6 provides a summary of all the differences found between these subtypes and obesity regarding the following variables: disgust sensitivity and aspects related to disgust, dissociation, and severity. Post hoc comparisons and their *p*-values are available in Table 7.

## 4. Discussion

Although various studies have explored the role of disgust in the onset and course of eating disorders, the exact role of disgust in different subtypes of eating disorders and obesity remains unclear. This study had several objectives: first, to explore the correlations between sensitivity to disgust, dissociative symptoms, and severity among participants; second, to investigate whether women of the clinical group exhibit higher sensitivity to disgust and greater dissociation compared to the non-clinical group; third, to examine which dimensions related to disgust are most prevalent in the clinical population; and finally, to analyze these differences within the clinical group by subtype.

Our results partially support our first hypothesis; disgust sensitivity is positively correlated with dissociative symptoms, but the correlations are not significant for severity when we analyze the correlations for each group. Based on global sensitivity to disgust, our results are consistent with previous studies [2,5,22,23,24,25,34], which found that sensitivity to disgust is high or higher in clinical populations, including those diagnosed with eating disorders. However, although, in our study, as in previous studies [34], a lower sensitivity to disgust was found in obese patients compared to lean patients, the sensitivity to disgust in patients with EDNOS in our study was lower than in obese patients. Disgust-based emotional reasoning is prevalent among those with weight and shape concerns, where feelings of disgust are interpreted as signals of potential weight gain, further complicating the emotional landscape surrounding obesity [59]. While disgust is often viewed negatively in the context of obesity, it can also serve as a protective mechanism against unhealthy behaviors. Understanding the dual nature of disgust—both as a potential barrier to healthy eating and as a response to societal stigma—can inform interventions aimed at improving emotional well-being in individuals with eating disorders and obesity.

Regarding dissociative symptoms, our findings align with previous studies [32,33,34]. The correlations observed suggest that high sensitivity to disgust is associated with greater dissociative symptoms, both in terms of compartmentalization and detachment. Among the samples analyzed, the group exhibiting the greatest severity, dissociation, and sensitivity to disgust was those diagnosed with anorexia nervosa. However, most of these studies have not explored the impact of the various dimensions of disgust on symptoms.

When evaluating the six dimensions of disgust according to the EMA [15]—hygiene, morality, sexuality, body transgression, animals and impairment/disease—we found significant differences in the dimensions of hygiene, sexuality and impairment/disease in the clinical group. These dimensions seem to play a crucial role in the psychopathology of eating disorders and obesity. Excessive concern with hygiene can intensify compulsive cleaning behaviors and a strict avoidance of “contaminated” foods. Regarding sexuality, rejection of sexual acts or bodies perceived as inappropriate can increase shame and body dissatisfaction, exacerbating social avoidance, especially in cases of anorexia. Finally, the impairment/disease dimension, associated with the fear of pathogens, can manifest itself in extreme restrictive behaviors and dietary rigidity to avoid foods considered “dangerous.” This pattern of avoidance and fear can aggravate the symptoms of eating disorders and complicate clinical intervention, since disgust is a difficult emotion to modify, contributing to resistance to treatment and the risk of relapse.

Furthermore, comparative analysis between ED subtypes and obesity showed that disgust sensitivity, symptom severity, and dissociation varied significantly between these groups. Anorexia nervosa had the highest levels in these variables, suggesting an emotional hypersensitivity that exacerbates food avoidance and disconnection with one’s own body. In binge eating disorder, the hygiene and impairment/disease dimensions were higher, which could reflect a negative perception of the body and a deep aversion to lack of control. On the other hand, people with obesity had the highest scores in the sexuality dimension, suggesting emotional conflicts related to self-image and the perception of the body as sexually inappropriate or unacceptable, which can aggravate feelings of shame and social avoidance. This specific disgust towards sexuality could contribute to greater isolation and low self-esteem, interfering with the ability to participate in healthy interpersonal relationships. Together, these differences underscore that disgust is not only present across the board in eating disorders and obesity, but manifests in ways that amplify the particular symptoms of each subtype.

According to Anderson et al. (2021) [5], the dimension of hygiene plays a significant role in eating disorders, influencing risk, maintenance, and aversive learning processes. Previous results suggest that disgust, particularly in its dimension of hygiene, plays a central role in avoidance eating disorders such as ARFID (Avoidant/Restrictive Food Intake Disorder), by influencing eating behaviors through the avoidance of potentially contaminated foods [33]. On the other hand, few studies specifically examine the sexual dimension of disgust in individuals with eating disorders. Most research focuses on the relationship between eating disorders and sexual function, concluding that disgust may play a role in this dimension [60,61]. Studies exploring the sexual dimension of disgust suggest that the connection between eating disorders and sexual disgust is associated with avoidance of sexual maturation and intimacy [62], and link anorexia to disgust toward bodily changes during puberty. It is also associated with possible histories of sexual victimization, which have been shown to relate both to eating disorders and dissociative symptoms, with sensitivity to disgust potentially being a mediating factor [63].

Eating disorders and the dimension of contamination/disease seem intrinsically related due to aversion to signs of illness, death, and decay [8], although there are not many studies specifically exploring this dimension in relation to eating disorders. In the context of eating disorders, this dimension of disgust appears in various forms; individuals with eating disorders often have a distorted perception of their bodies, viewing them as “contaminated” or “imperfect”. This perception may be influenced by the impairment/disease dimension of disgust, where any perceived sign of “imperfection”, such as body fat or loose skin, is associated with impairment and illness, eliciting a strong repulsion toward their own bodies [5]. Additionally, aversion to signs of impairment and disease can lead to extreme purging behaviors (such as self-induced vomiting) and dietary restrictions. The disgust toward a body that does not meet ideal standards may motivate these destructive behaviors. Furthermore, disgust associated with this dimension may manifest in the avoidance of certain foods considered “unhealthy” or “contaminating”. Individuals with eating disorders and obesity may develop rigid dietary rules to avoid foods they perceive as contributing to bodily deterioration.

Regarding dissociative symptoms, our study found statistically significant differences both between groups and between different subtypes of eating disorders and obesity. It has been proposed that dissociation may function as a coping mechanism for contradictions and intolerable perceptions [37]. Some studies link dissociative symptomatology as a mediating factor between psychological trauma and eating disorders [37], and obese people often experience high levels of dissociation linked to childhood trauma [64]. On the other hand, strong aversion to one’s perceived impaired body may lead to dissociative mechanisms, where the individual mentally distances themselves from their bodily sensations and experiences. Furthermore, the relationship between dissociation and body image issues suggests that dissociative symptoms may impair the ability to accurately perceive one’s body, further complicating eating disorder symptoms [65] and potentially exacerbating obesity [66]. Finally, the microbiome–gut–brain axis plays a crucial role in obesity, with dissociation potentially disrupting this communication pathway. A failure to establish a healthy microbiome–gut interface can lead to non-communicable diseases, including obesity [67]. These results raise the possibility that disgust may be a relevant emotion in some dissociative responses and this opens a possibility of understanding and addressing this type of phenomena.

As limitations, it is worth highlighting the study design, small sample size in general, and concretely, the sample size for obesity was limited, and results in this study might change if the number of participants were increased, allowing for more relevant comparisons. On the other hand, it is worth mentioning that, although therapists were told that participants should have an eating disorder as their primary diagnosis and not have significant comorbidities, these were not explicitly recorded. Additionally, considering the results in the non-clinical population regarding suicidal ideation, it is possible that the sample may have limitations in representing the general population. Various studies [68,69,70,71] indicate that the prevalence of suicidal ideation in the general population is highly variable, even exceeding 50% depending on factors such as culture and context. Another limitation is the heterogeneity of measurement instruments used in different studies to assess sensitivity to disgust or aspects associated with this emotion. Finally, it is worth mentioning that it may be interesting to confirm the diagnosis of TCA with a diagnostic test.

## 5. Conclusions

In conclusion, sensitivity to disgust and certain dimensions of disgust, such as hygiene, sexuality, and impairment/disease, may play a significant role in the etiology and maintenance of eating disorders. Furthermore, there is a significant relationship between the emotion of disgust and dissociative symptomatology, suggesting that this interaction may influence the severity and clinical presentation of each subtype of eating disorder. A better understanding of this relationship could have a direct impact on the design of treatments better suited to each clinical profile, facilitating interventions that address both disgust and dissociative mechanisms. Addressing disgust in the treatment of eating disorders could enhance the effectiveness of therapeutic interventions, facilitating the reintroduction of foods and body acceptance through techniques such as exposure and desensitization. This underscores the importance of considering disgust in the treatment of these disorders. These factors should be taken into account, and it should be studied in more detail in future research on the topic. Further research into the factors that contribute to both the onset and maintenance of eating disorders and obesity is critical. This will allow for the development of more effective prevention and treatment strategies based on a more detailed understanding of the mechanisms underlying these pathologies.

## Figures and Tables

**Table 1:** Six basic dimensions of disgust.

Disgust Dimension	Definition
Hygiene	The hygiene dimension of disgust is related to the aversion to dirt, contamination, and waste. This includes repulsion toward elements such as garbage, excrement, bodily fluids, and foul odors [27]. From an evolutionary perspective, this dimension may have developed to avoid exposure to pathogens and maintain health.
Moral	The moral dimension of disgust refers to the emotional reaction to behaviors and actions that violate ethical and social norms [28]. From a psychological perspective, moral disgust helps maintain social cohesion and moral standards within a community.
Sexual	The sexual dimension of disgust is associated with aversion to acts or sexual stimuli that are considered inappropriate or repulsive [29]. Psychologically, this type of disgust may serve to protect physical and emotional integrity, as well as to regulate sexual behavior in line with social and cultural norms.
Body transgression	The body transgression dimension refers to aversion to violations of bodily integrity. This includes reactions to wounds, mutilations, visible infections, and deformities. This dimension of disgust may be linked to an evolutionary response to avoid contact with elements that can cause physical harm or transmit diseases.
Animals	The dimension of disgust related to animals [30] focuses on the repulsion towards certain animals, especially those that may transmit diseases or cause harm. From a psychological perspective, this aversion may have evolved as a protective mechanism to avoid exposure to pathogens.
Impairment/disease	The impairment/disease dimension is related to aversion to signs of disease, death, and decomposition [6,24]. This includes disgust reactions to decomposing bodies, people with visible diseases, or symptoms of disease. Psychologically, this dimension of disgust may be an evolutionary mechanism to avoid contagion and protect health. Each of these dimensions of disgust has significant implications for understanding human emotions and their role in adaptive behavior, social regulation, and mental health

**Table 2:** The main sociodemographic characteristics of the clinical group (mean and standard deviation).

Variables	AN (N = 31)	BN (N = 25)	BED (N = 37)	Obesity (N = 14)	EDNOS (N = 40)	Clinical Group (N = 147)	*p*
Age	37.82 (11.18)	34.84 (8.46)	37.41 (10.12)	44.21 (9.01)	35.73 (9.29)	35.45 (9.35)	0.002
Weight (kg)	53.10 (12.18)	64.73 (18.21)	75.68 (19.83)	94.15 (19.42)	68.75 (17.02)	69.28 (20.32)	0.000
Height (cm)	165.04 (5.86)	164.63 (8.23)	165.24 (7.2)	163.38 (4.5)	163.30 (6.04)	164.33 (6.23)	0.692
Disorder duration	13.8 (9.5)	16.50 (8.23)	9.42 (7.34)	15.67 (10.01)	15.13 (8.04)	13.65 (8.71)	0.108
Nº Hospitalizations	2.63 (6.02)	0.52 (1.17)	0.6 (0.23)	0 (0)	0.85 (1.74)	0.84 (2.97)	0.000
Suicidal ideation	0.68 (0.48)	0.68 (0.47)	0.51 (0.51)	0.46 (0.52)	0.57 (0.5)	0.59 (0.5)	0.469
Self-injurious behaviors	0.94 (0.25)	0.72(0.46)	0.43 (0.5)	0.36 (0.5)	0.63 (0.5)	0.63 (0.49)	0.000
Suicide attempts	0.42(0.5)	0.3 (0.47)	0.16 (0.37)	0.15 (0.38)	0.24 (0.4)	0.23 (0.42)	0.201
DCI-Total	76.46 (46)	63.08 (32.57)	59.78 (35.72)	46.53 (32.2)	51.54 (40.62)	58.65 (39.44)	0.129
EMA-Total	61.25 (18.88)	51.83 (13.53)	59.81 (18.39)	60 (16.32)	55.09 (20.92)	57.15 (18.06)	0.277

**Table 3:** Pearson correlations between disgust sensitivity and dissociative symptoms of detachment and compartmentalization, and severity across the control group.

Variables	DCI-Detachment	DCI-Compartmentalization	DCI-Total	Severity
EMA-Hygiene	0.21 **	0.12	0.17 *	0.4
EMA-Moral	0.16 *	0.11	0.14	0.36
EMA-Sexual	0.20 *	0.09	0.16	0.39
EMA-Body Transgression	0.00	−0.05	−0.02	−0.00
EMA-Animals	0.01	−0.12	−0.58	0.03
EMA-Impairment/Disease	0.10	0.07	0.10	0.3
EMA-Total	0.18 *	0.05	0.13	0.35

* *p* < 0.05. ** *p* < 0.01.

**Table 4:** Pearson correlations between disgust sensitivity and dissociative symptoms of detachment and compartmentalization, and severity across the clinical group.

Variables	DCI-Detachment	DCI-Compartmentalization	DCI-Total	Severity
EMA-Hygiene	0.29 **	0.23 **	0.26 **	0.14
EMA-Moral	0.33 **	0.28 **	0.30 **	−0.01
EMA-Sexual	0.24 **	0.21 **	0.22 **	0.20
EMA-Body Transgression	0.08	0.18	0.11	0.12
EMA-Animals	0.14	0.05	0.10	0.21
EMA-Impairment/Disease	0.14	0.18 *	0.18 *	0.11
EMA-Total	0.33 **	0.29 **	0.32 **	0.21

* *p* < 0.05. ** *p* < 0.01.

**Table 5:** Differences between the clinical group and the control group for the variables: disgust sensitivity and aspects related to disgust emotion, dissociation, and severity, including effect size.

Variables	Clinical Group Mean (SD)	Control Group Mean (SD)	*p*	Cohen’s d
EMA-Hygiene	11.11 (4.61)	9.23 (3.94)	0.000	0.44
EMA-Moral	11.05 (5.36)	10.27 (5.08)	0.184	0.15
EMA-Sexual	8.98 (5.43)	7.09 (4.94)	0.001	0.36
EMA-Body Transgression	7.01 (5.05)	7.39 (5.07)	0.515	−0.08
EMA-Animals	11.69 (4.78)	10.26 (4.22)	0.007	0.32
EMA-Impairment/Disease	6.35 (4.46)	4.56 (3.43)	0.000	0.45
EMA-Total	57.15 (18.06)	48.82 (17.01)	0.000	0.47
DCI-Detachment	29.15 (18.78)	15.31 (14.36)	0.000	0.83
DCI-Compartmentalization	22.60 (19.49)	6.86 (12.03)	0.000	0.97
DCI-Total	58.65 (39.44)	27.02 (26.41)	0.000	0.94
CGI-Severity	3.32 (1.8)	2.18 (2.45)	0.002	0.53

**Table 6:** Differences among eating disorder subtypes and obesity in terms of disgust sensitivity and disgust-related aspects, dissociation, and severity.

Variables	AN Mean (SD)	BN Mean (SD)	BED Mean (SD)	Obesity Mean (SD)	EDNOS Mean (SD)	*p*
EMA-Hygiene	11 (5.16)	10.36 (3.74)	12.08 (4.96)	12 (3.44)	10.3 (4.61)	0.015
EMA-Moral	10.80 (5.13)	10.56 (5.33)	12.7 (4.89)	11.71 (5.96)	9.22 (5.85)	0.095
EMA-Sexual	10.32 (5.29)	7.48 (5.07)	8.4 (4.89)	11.28 (6.67)	9.32 (5.93)	0.007
EMA-Body Transgression	7.94 (5.61)	6.84 (5.03)	7.86 (5.19)	8.07 (4.28)	5.85 (5.17)	0.117
EMA-Animals	12.41 (4.56)	11.22 (4.23)	11.37 (5.52)	10.13 (3.44)	11.88 (5.31)	0.189
EMA-Impairment/Disease	6.58 (4.54)	5.08 (3.17)	7.37 (4.70)	6.69 (3.54)	6.79 (5.42)	0.001
EMA-Total	61.25 (18.88)	51.83 (13.53)	59.81 (18.89)	60 (16.32)	55.09 (20.92)	0.002
DCI-Detachment	37.04 (20.87)	30.52 (16.54)	29.18 (17.44)	24.46 (15.14)	26.03 (19.01)	0.000
DCI- Compartmentalization	31 (22.4)	23.96 (17.23)	23.56 (18.41)	16.30 (17.08)	19.21 (19.66)	0.000
DCI-Total	76.45 (45.95)	63.08 (32.57)	59.78 (35.72)	46.53 (32.52)	51.54 (40.62)	0.000
CGI-Severity	4.06 (1.43)	3.56 (1.58)	3 (1.68)	2.90 (1.85)	3.05 (1.68)	0.001

**Table 7:** Significant pairwise comparisons between variables using Dunn’s test.

Variables	Pairwise Comparisons	N	Statistical Test	Error dev.	Statistical Test dev.	Sig.	Adjusted Sig.
DCI- Detachment	Women without ED *-EDNOS	Control:158 EDNOS:40	−57.42	17.43	−3.29	0.001	0.021
	Women without ED-BED	Control:158 BED:37	−80.71	16.65	−4.84	0.000	0.000
	Women without ED-BN	Control:158 BN:25	−84.77	20.29	−4.17	0.001	0.001
	Women without ED-AN	Control:158 AN:31	−103.74	19.92	−5.20	0.000	0.000
DCI-Compartmentalization	Women without ED-EDNOS	Control:158 EDNOS:40	−76.65	17.31	−4.42	0.000	0.020
	Women without ED-Obesity	Control:158 Obesity:14	−82.85	26.00	−3.18	0.001	0.030
	Women without ED-BN	Control:158 BN:25	−104.43	16.53	−6.31	0.000	0.000
	Women without ED-BED	Control:158 BED:37	−100.86	20.15	−5.00	0.000	0.000
	Women without ED-AN	Control:158 AN:31	−115.37	19.79	−5.82	0.000	0.000
DCI- Total	Women without ED-EDNOS	Control:158 EDNOS:40	−64.73	17.44	−3.71	0.000	0.004
	Women without ED-BN	Control:158 BN:25	−97.77	16.65	−5.87	0.000	0.000
	Women without ED-BED	Control:158 BED:37	−97.60	20.29	−4.80	0.000	0.000
	Women without ED-AN	Control:158 AN:31	−112.27	19.93	−5.63	0.000	0.000
EMA-Hygiene	Women without ED-BED	Control:158 BED:37	−53.72	17.51	−3.06	0.002	0.045
EMA-Sexual	Women without ED-AN	Control:158 AN:31	−61.38	18.82	−3.26	0.001	0.023
EMA-Impairment/Disease	Women without ED-BED	Control:158 BED:37	−61.30	16.57	−3.69	0.000	0.005

* Women without ED constitute the control group.

## Data Availability

Data are contained within the article.
